# Influence of concurrent chemotherapy on locoregionally advanced nasopharyngeal carcinoma treated with neoadjuvant chemotherapy plus intensity-modulated radiotherapy: A retrospective matched analysis

**DOI:** 10.1038/s41598-020-59470-w

**Published:** 2020-02-12

**Authors:** Fangzheng Wang, Chuner Jiang, Lai Wang, Fengqin Yan, Quanquan Sun, Zhimin Ye, Tongxin Liu, Zhenfu Fu, Yangming Jiang

**Affiliations:** 1 Institute of Cancer and Basic Medicine (ICBM), Chinese Academy of Sciences, Zhejiang, Hangzhou 310022 People’s Republic of China; 2 Department of Radiation Oncology, Cancer Hospital of the University of Chinese Academy of Sciences, Zhejiang, Hangzhou 310022 People’s Republic of China; 3 Department of Radiation Oncology, Zhejiang Cancer Hospital, Zhejiang, Hangzhou 310022 People’s Republic of China; 4 Key Laboratory of Radiation Oncology of Zhejiang Province, Zhejiang, Hangzhou 310022 People’s Republic of China; 5 Department of Breast Tumor Surgery, Cancer Hospital of the University of Chinese Academy of Sciences, Zhejiang, Hangzhou 310022 People’s Republic of China; 6 Department of Breast Tumor Surgery, Zhejiang Cancer Hospital, Zhejiang, Hangzhou 310022 People’s Republic of China; 7 Department of Didital Earth, Institute of Remote Sensing and Digital Earth, CAS, Beijing, 100101 People’s Republic of China

**Keywords:** Cancer therapy, Head and neck cancer

## Abstract

Neoadjuvant chemotherapy (NAC) combined with intensity-modulated radiotherapy (IMRT) plus concurrent chemotherapy (CC) will be the new standard treatment for locoregionally advanced nasopharyngeal carcinoma (NPC) patients. However, many patients fail to receive CC for multiple reasons. We aimed to investigate long-term survival outcomes and toxicities in these patients with NPC treated with additional NAC plus concurrent chemoradiotherapy (CCRT) or IMRT alone. In total, 1,378 previously untreated, newly diagnosed locoregionally advanced NPC patients receiving NAC plus IMRT with or without CC were retrospectively reviewed. We used a propensity score-matched (PSM) method with 1:1 matching to identify paired patients according to various covariates. Survival outcomes and toxicities were compared between the two groups. In total, 288 pairs were identified. With a median follow-up of 86 (range: 8–110) months, the estimated 5-year locoregional relapse-free survival, distant metastasis-free survival, progression-free survival (PFS), and overall survival rates in patients treated with NAC plus CCRT vs. NAC plus IMRT alone were 96.1% vs. 94.7% (P = 0.201), 93.7% vs. 89.8% (P = 0.129), 91.3% vs. 85.1% (P = 0.024), and 93.0% vs. 90.6% (P = 0.362), respectively. Multivariate analysis showed that CC omission was a prognostic factor for worse PFS. In a subgroup analysis, PFS did not differ significantly between two groups of female patients or aged <60 years or stage T1–2 or stage N0-1 disease. However, fewer acute complications were observed in the NAC plus IMRT alone group. NAC with IMRT alone confers similar survival rates and less acute toxicities. Specifically, NAC plus IMRT alone may be enough for female patients <60 years with stage T1-2 or stage N0-1. However, a prospective randomised trial is needed to validate these results.

## Introduction

Nasopharyngeal carcinoma (NPC) is a unique type of head and neck cancer with the cute incidence of 15–50 cases per 100,000 individuals annually in endemic regions, such as southern China, Singapore, and Malaysia^1^. In some endemic areas, the incidence and mortality rates for NPC have reduced due to lifestyle changes^2,3^. Nevertheless, NPC is still one of the leading causes of cancer death, with a global mortality rate of about 50,000 individuals per year^1,4^.

Because of the high sensitivity to radiation and the complicated anatomical structure, radiotherapy (RT) is the primary treatment for NPC. Approximately 60–70% of patients present with locoregionally advanced NPC at the time of diagnosis^5^. Previous studies showed that when compared with two-dimension RT, intensity-modulated radiation therapy (IMRT) provided benefit for locoregional control, while it did not prolong survival outcomes or reduced distant failure^6,7^. Al-Sarraf *et al*. performed a meta-analysis to demonstrate that a combination of RT and chemotherapy improved 4% to 6% in 5-year survival and reduced mortality by 18%^8^. Concurrent chemoradiotherapy (CCRT) with or without adjuvant chemotherapy (AC) is beneficial in overall survival (OS) and has become the standard treatment for locoregionally advanced NPC, even with the acute toxicities^9–11^. A previous meta-analysis showed that when compared with CCRT alone, the addition of neoadjuvant chemotherapy (NAC) before CCRT reduced distant failure in locoregionally advanced NPC patients^12,13^. A different meta-analysis showed that NAC before to CCRT significantly improved progression-free survival (PFS) and OS^14^. However, the efficacy of NAC followed by CCRT in patients with locoregionally advanced NPC remains controversial^15–17^. Considering these results, the addition of NAC before CCRT has been a promising therapeutic alternative for locoregionally advanced NPC patients in the era of IMRT.

Unfortunately, a few patients do not receive concurrent chemotherapy (CC) due to multiple reasons. The reasons include treatment-associated toxicities, economic conditions, refusal without due cause, and so on. It remains unclear whether adding NAC before IMRT alone is inferior to CCRT with or without NAC. Komatsu *et al*. compared CCRT with NAC plus RT and observed a similar survival between two arms^18^. Huang *et al*. performed a randomised study to compare the efficacy of NAC plus CCRT versus NAC plus RT for NPC patients and found that NAC plus RT was not inferior to NAC plus CCRT in OS and PFS^17^. Wu *et al*. also indicated that NAC before RT provided long-term outcomes similar to those with CCRT for locoregionally advanced NPC^19^. A phase III randomised study by Xu *et al*. showed that NAC added to IMRT plus AC yielded similar OS and PFS with CCRT plus AC, while with less acute toxicities^20^. The 2-dimension RT technology was used in the above studies. However, IMRT showed greater improvement in survival than 2-dimension RT^21,22^. Based on these encouraging results, the addition of NAC to IMRT alone may be a promising option with encouraging outcomes and less toxicity. Few studies on the safety and efficacy of NAC plus IMRT alone in locoregionally advanced NPC have been reported. Therefore, we performed a retrospective, matched analysis of locoregionally advanced NPC patients to compare the efficacy and safety of adding NAC to IMRT alone with NAC before CCRT.

## Results

### Patient characteristics

From May 2008 through April 2014, 1,378 previously untreated patients diagnosed with locoregionally advanced NPC received NAC plus IMRT with or without CC. Among these patients, 1078 patients were treated with additional NAC to CCRT, and 300 patients received NAC, followed by IMRT alone. After matching, there were no statistically significant differences in age, sex, T-stage, N-stage, clinical stage, NAC regimen, and NAC cycle between two groups. Table 1 listed the baseline characteristics of matched patients.

**Table 1:** Baseline characteristic of 576 patients with locoregionally advanced NPC.

Characteristic	NAC + CCRT	NAC + IMRT	P
**Sex**			
Male	190	177	0.260
Female	98	111	
**Age (years)**			
Range	16–77	13–80	
Median	49	50	
<60	233	218	0.129
≥60	55	70	
WHO pathology			0.842
Type I	2	3	
Type II	15	13	
Type III	271	272	
T stage*			0.944
T1	17	17	
T2	54	58	
T3	136	129	
T4	81	84	
N stage*			0.360
N0	17	20	
N1	57	70	
N2	175	154	
N3	39	44	
Clinical stage*			0.785
III	177	169	
IVA	71	75	
IVB	40	44	
**AC**			
No	107	120	0.268
Yes	181	168	
NAC regimens			0.343
TPF	56	52	
TP	36	49	
GP	9	13	
FP	187	174	
**Cycle of NAC**			
<2	157	143	0.243
≥2	131	145	

WHO: World Health Organization. AC: adjuvant chemotherapy; NAC: neoadjuvant chemotherapy; TPF docetaxel/cisplatin/fluorouracil; TP docetaxel/cisplatin, GP gemcitabine/cisplatin; PF cisplatin/fluorouracil.

*The 7th AJCC/UICC staging system.

### Survivals

**Figure 1:**
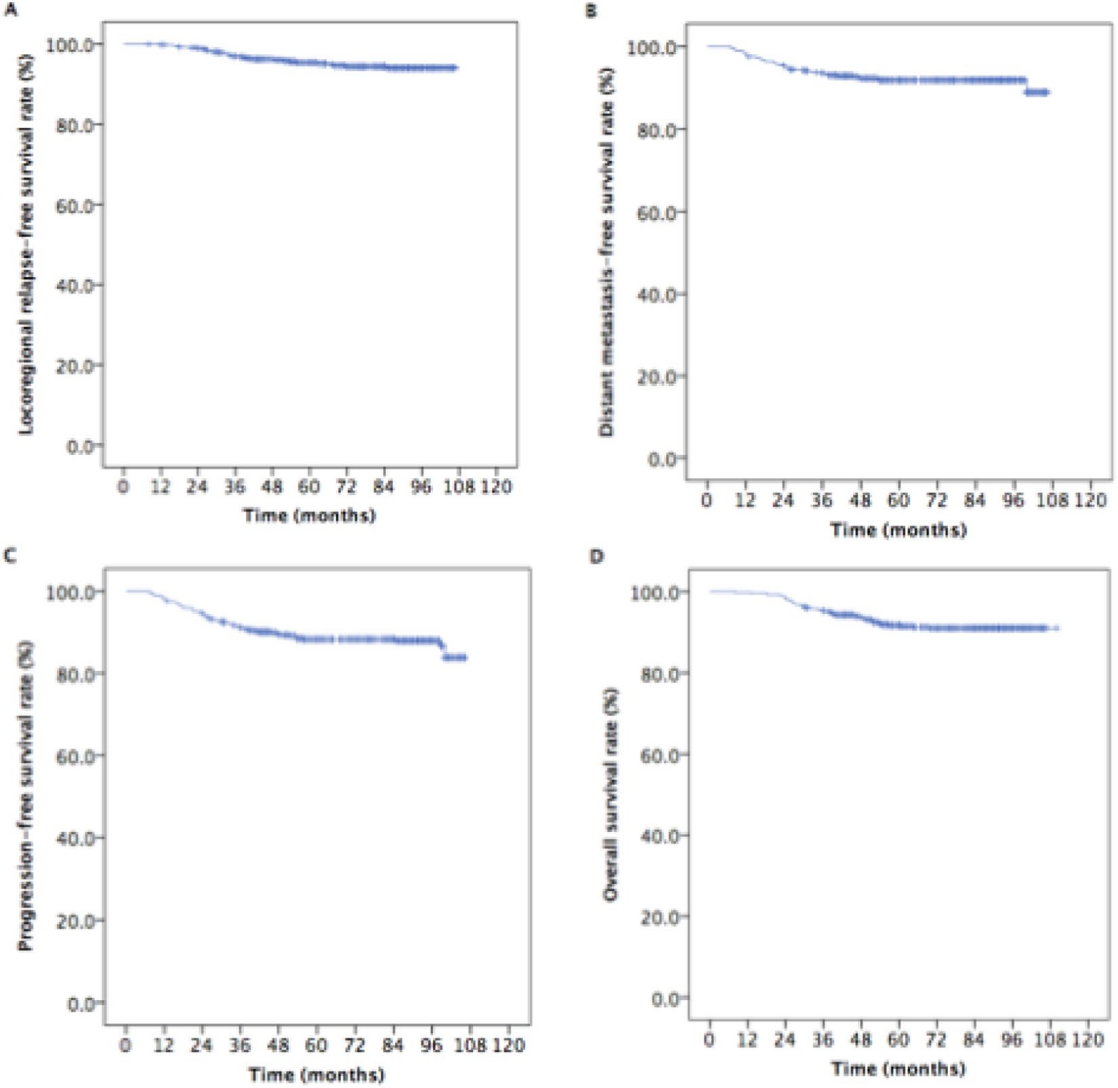
Kaplan-Meier estimates of the survival in 576 patients with locoregionally advanced nasopharyngeal carcinoma.

With a median follow-up duration of 86 months (range, 8–110 months), the 5-year estimated rates in loco-regional relapse-free survival (LRRFS), distant metastasis-free survival (DMFS), PFS, and OS for the entire cohort of patients were 95.4%, 91.8%, 88.2%, and 91.7%, respectively. Figure 1 provided the Kaplan-Meier curves of the survival in these patients.

**Figure 2:**
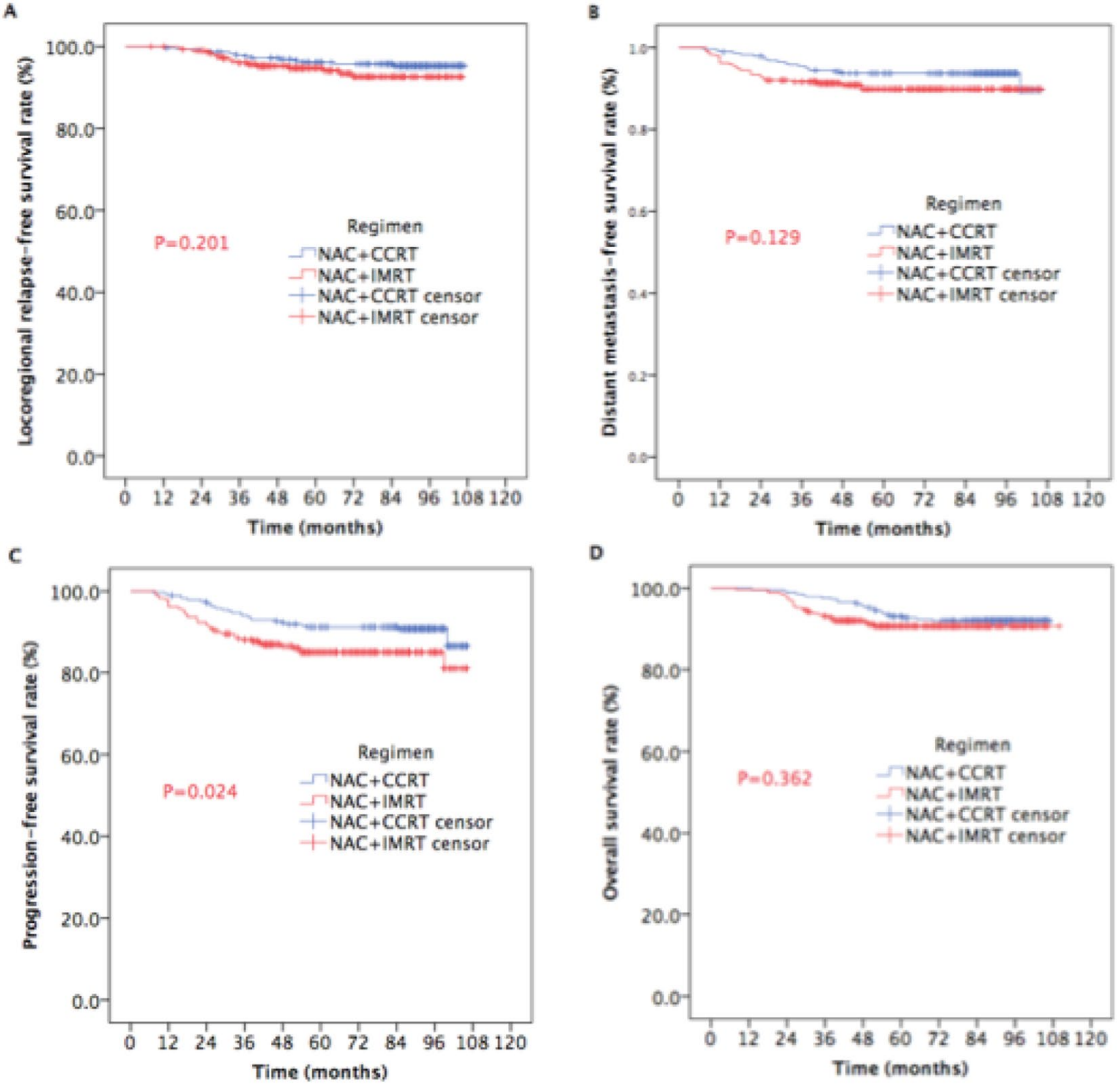
Kaplan-Meier estimates of the survival in NPC patients receiving NAC plus IMRT with or without CC.

Statistically significant differences in LRRFS, DMFS, and OS were not found between the two groups (Fig. 2A: 5-year LRRFS: 96.1% vs. 94.7%, respectively, P = 0.201. Figure 2B: 5-year DMFS: 93.7% vs. 89.8%, respectively, P = 0.129. Figure 2D: 5-year OS: 93.0% vs. 90.6%, respectively, P = 0.362.) The 5-year PFS rate was significantly higher for patients treated with additional NAC before CCRT than for those treated NAC before IMRT alone (Fig. 2C: 91.3% vs. 85.1%, respectively; P = 0.024).

### Patterns of treatment failure

Of all participants, 70 patients (12.2%) experienced “any” treatment failure during the last follow-up. In the NAC + CCRT group, 28 patients (9.7%) experienced “any” failure (locoregional relapse only occurred in 13 patients, distant metastases was observed in 15 patients, both locoregional and distant failure were found in five patients). In the NAC + IMRT group, 42 patients (14.9%) experienced “any” failure (locoregional recurrence was developed in 14 patients, distant metastases occurred in 25 patients, both locoregional and distant failure were observed in three patients). The details are summarised in Table 2. The median time to failure between the two groups was 92 months (range 9 to 106 months) and 61 months (range 8 to 106 months), respectively.

**Table 2:** Site and incidence of treatment failure.

Sites	NAC + CCRT	NAC + IMRT alone	P
	N = 288	N = 288	
Locoregional only	13	14	0.336
Locoregional and distant	5	3	
Distant only	15	25	
Non-failure	260	246	

### Prognostic factors

We assessed the potential prognostic factors associated with LRRFS, DMFS, PFS, and OS by univariate and multivariate regression analysis models. Age, sex, T category, N category, clinical stage, NAC regimen, NAC cycle, AC, and treatment regimen were analysed as the possible prognostic factors for the NPC patients enrolled in the current study. On the univariate analysis, we found that T3-4 and IVA/B were significantly associated with poorer OS. And the 5-year DMFS and PFS of patients with clinical stage III were superior to those with IVA/B, but there were no statistically significant differences. While 5-year PFS in patients treated with NAC plus IMRT alone was worse than those receiving the addition of NAC to CCRT. The results of 576 patients with locoregionally advanced NPC by using univariate analysis are summarised in Table 3.

**Table 3:** Prognostic factors on survival outcomes of 576 NPC patients using univariate analysis.

Characteristics	n	OS (%)	P	LRRFS (%)	P	DMFS (%)	P	PFS (%)	P
Age			0.231		0.748		0.318		0.860
<60	451	92.3		95.4		91.3		88.0	
≥60	125	89.6		95.3		93.4		88.9	
Sex			0.603		0.141		0.305		0.110
Male	367	91.2		94.8		91.1		86.8	
Female	209	92.6		96.9		93.2		90.7	
T category			0.006		0.548		0.623		0.327
T1–2	146	93.0		96.3		93.1		90.7	
T3-4	430	91.2		95.0		91.3		87.3	
N category			0.360		0.548		0.623		0.327
N0-1	164	92.9		96.3		93.1		90.7	
N2-3	412	91.2		95.0		91.3		87.3	
Clinical stage			0.008		0.356		0.056		0.080
III	347	94.4		95.7		93.2		89.6	
IVA/B	230	87.6		94.9		89.7		86.1	
NAC regimen			0.683		0.692		0.579		0.730
TPF	108	94.1		91.6		95.4		89.5	
TP	85	92.3		97.0		92.2		89.3	
GP	22	90.9		95.0		85.9		81.3	
PF	361	96.4		95.8		91.1		87.9	
NAC cycle			0.719		0.656		0.201		0.693
<2	300	91.4		96.0		89.8		87.1	
≥2	276	91.9		94.9		93.6		89.3	
AC			0.417		0.703		0.194		0.445
No	227	92.4		94.5		93.9		89.9	
Yes	349	91.2		96.0		90.4		87.1	
Regimen			0.362		0.201		0.129		0.024
NAC + IMRT	288	90.6		94.7		89.8		85.1	
NAC + CCRT	288	93		96.1		93.7		91.3	

Abbreviations: LRRFS locoregional relapse-free survival, DMFS distant metastases-free survival, PFS progression-free survival, OS overall survival, NAC neoadjuvant chemotherapy, AC adjuvant chemotherapy, CCRT concurrent chemoradiotherapy, IMRT intensity-modulated radiotherapy, TP docetaxel/cisplatin, TPF docetaxel/cisplatin/fluorouracil, GP gemcitabine/cisplatin, FP cisplatin/fluorouracil.

For the multivariate analysis, we selected age (<60 years vs. ≥60 years), sex (male vs. female), clinical stage (III vs. IVA-B), regimen (NAC + CCRT vs. NAC + IMRT alone), AC (Without vs. With), NAC cycle (<2 vs. ≥2). The treatment regimen was not an independent predictor of LRRFS, DMFS, and OS. NAC + IMRT alone was associated with poorer prognostic outcomes for PFS (HR = 0.541, 95% CI = 0.332–0.884, P = 0.014). IVA/B was an adverse independent prognostic factor of DMFS and OS (P = 0.044 and P = 0.009, respectively) Results in Table 4.

**Table 4:** Summary of multivariate analyses of prognostic factors in the 576 NPC patients.

Endpoint	Factor	HR	95% CI	P
LRRFS	Age: <60 years vs. ≥60 years	0.975	0.408–2.386	0.954
	Sex: male vs. female	1.872	0.799–4.386	0.149
	Clinical stage: III vs. IVA-B	0.757	0.364–1.573	0.455
	Regimen: NAC + IMRT vs. NAC + CCRT	0.613	0.293–1.284	0.195
	AC: No vs. Yes	1.114	0.504–2.461	0.790
	NAC cycle: <2 vs. ≥2	1.013	0.455–2.254	0.975
DMFS	Age: <60 years vs. ≥60 years	1.539	0.704–3.364	0.280
	Sex: male vs. female	1.356	0.723–2.543	0.342
	Clinical stage: III vs. IVA-B	0.552	0.309–0.985	0.044
	Regimen: NAC + IMRT vs. NAC + CCRT	0.565	0.314–1.017	0.057
	AC: No vs. Yes	0.831	0.424–1.628	0.589
	NAC cycle: <2 vs. ≥2	1.675	0.888–3.161	0.111
PFS	Age: <60 years vs. ≥60 years	1.130	0.627–2.039	0.684
	Sex: male vs. female	1.525	0.897–2.593	0.120
	Clinical stage: III vs. IVA-B	0.665	0.412–1.072	0.094
	Regimen: NAC + IMRT vs. NAC + CCRT	0.541	0.332–0.884	0.014
	AC: No vs. Yes	0.880	0.514–1.508	0.642
	NAC cycle: <2 vs. ≥2	1.263	0.748–2.130	0.382
OS	Age: <60 years vs. ≥60 years	0.685	0.362–1.295	0.244
	Sex: male vs. female	1.068	0.585–1.954	0.828
	Clinical stage: III vs. IVA-B	0.461	0.258–0.824	0.009
	Regimen: NAC + IMRT vs. NAC + CCRT	0.764	0.431–1.354	0.357
	AC: No vs. Yes	0.755	0.396–1.440	0.396
	NAC cycle: <2 vs. ≥2	1.218	0.658–2.254	0.394

Abbreviations: LRRFS locoregional relapse-free survival, DMFS distant metastasis-free survival, PFS progression-free survival, OS overall survival, NAC neoadjuvant chemotherapy, AC adjuvant chemotherapy, CCRT concurrent chemoradiotherapy, IMRT intensity-modulated radiotherapy.

### Subgroup analysis

**Figure 3:**
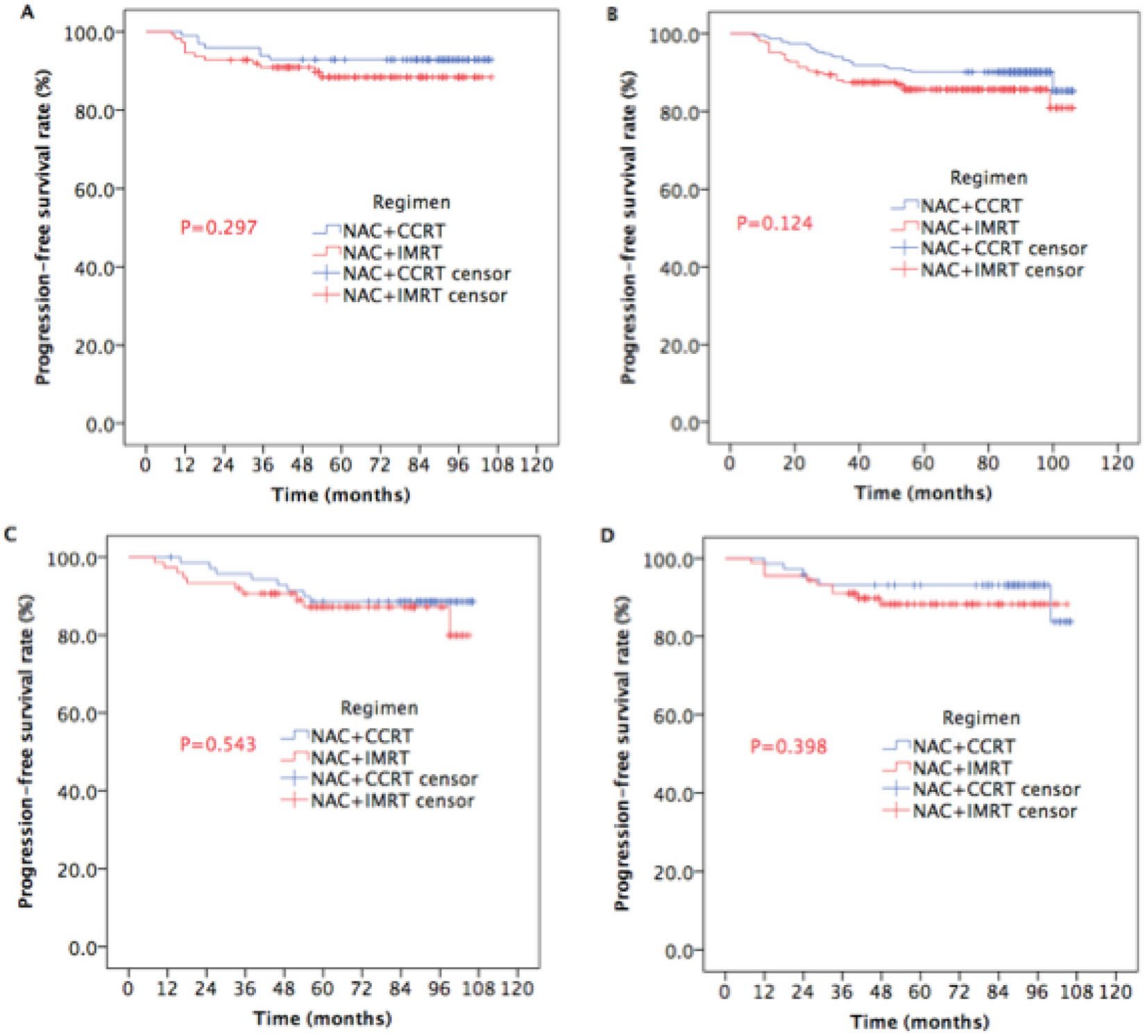
Kaplan-Meier estimates of PFS in subgroup NPC patients receiving NAC plus IMRT with or without CC.

The subgroup analysis allowed us to identify the patients who would benefit from NAC + CCRT in PFS according to age, sex, T category, and N category. For female patients, or <60 years, or T1-2 or N0-1, the 5-year PFS (92.9% vs. 88.4%, P = 0.297, Fig. 3A; 90.1% vs. 85.6%, P = 0.124, Fig. 3B; 88.6% vs. 87.2%, P = 0.543, Fig. 3C; 93.2% vs. 88.3%, P = 0.398, Fig. 3D) were comparable between the two groups.

**Figure 4:**
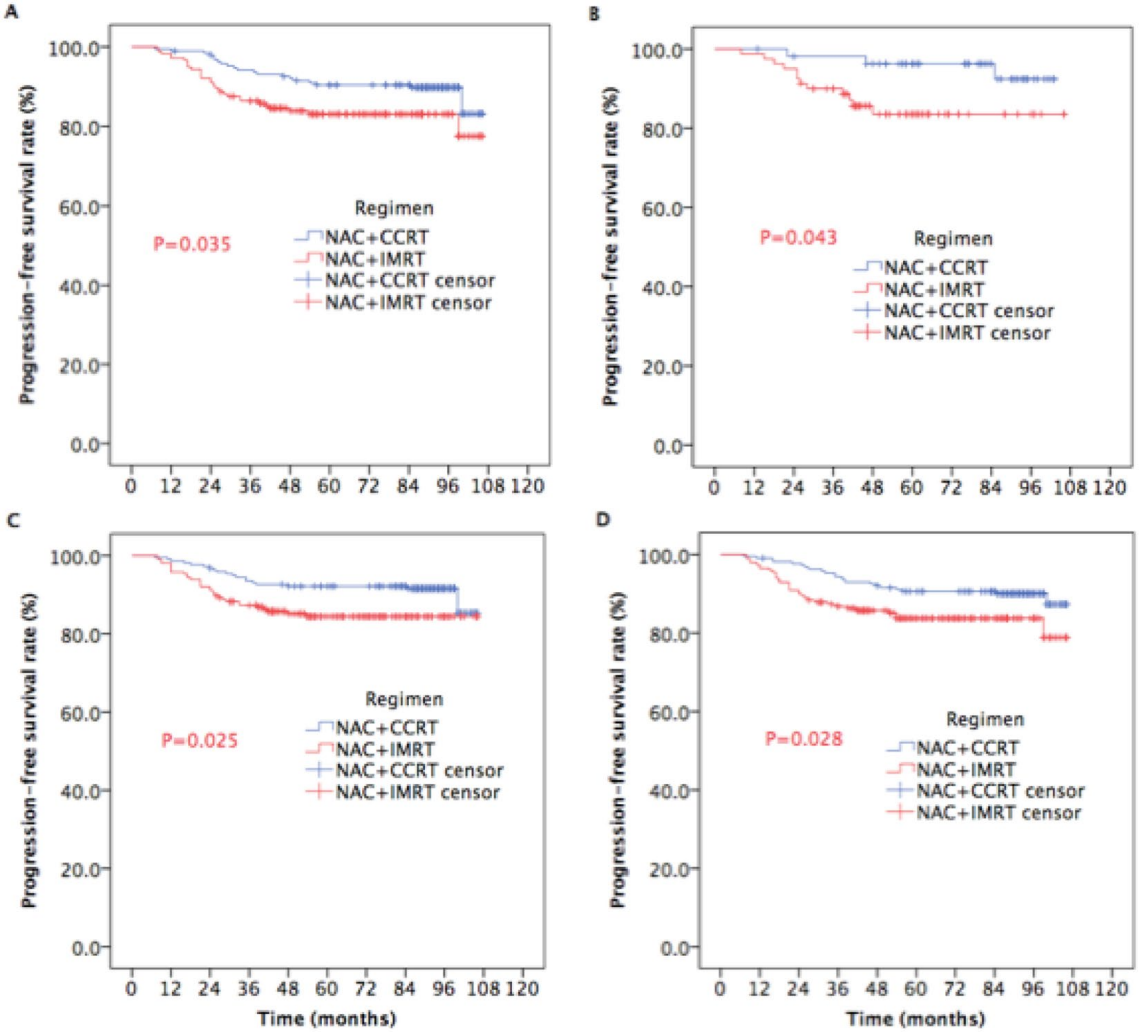
Kaplan-Meier estimates of PFS in subgroup NPC patients receiving NAC plus IMRT with or without CC.

Likewise, for male patients, or ≥60 years, or T3-4 or N2-3, adding NAC prior to CCRT significantly improved the 5-year PFS (90.4% vs. 83.1%, P = 0.033, Fig. 4A; 96.3% vs. 83.5%, P = 0.043, Fig. 4B; 92.1% vs. 84.4%, P = 0.025, Fig. 4C; 90.6% vs. 83.8%, P = 0.028, Fig. 4D), and there were statistically significant differences between two groups.

### Safety and toxicity

The treatment-related acute haematologic and non-haematologic adverse events during NAC and IMRT were retrospectively assessed using terms from the Common Terminology Criteria for Adverse Events (CTCAE v4.0). The profile of the most common side effect was listed in Table 5. The incidences of grade 3 to 4 acute toxicities in the NAC + IMRT group during NAC were similar to those in the NAC + CCRT group. During the IMRT period, grade 3/4 leukopenia and neutropenia in patients treated with NAC + CCRT vs. NAC + IMRT alone were reported in 67 (23.3%) vs. 40 (13.9%), and 59 (20.5%) vs. 39 (13.5%) patients respectively. The rates of grade 3/4 mucositis and nausea/vomiting between the two groups were 26.4% (76/288) vs. 13.9% (40/288) and 15.6% (45/288) vs. 2.4 (7/288), respectively. There were statistically significant differences seen (P < 0.05).

**Table 5:** Profile of toxicities during NAC and IMRT between two groups.

Adverse events	During NAC					During IMRT					
	NAC + CCRT		NAC + IMRT		P	NAC + CCRT		NAC + IMRTcancer incidence, mortalitycancer incidence, mortality			P
	0–2	3–4	0–2	3–4		0–2	3–4		0–2	3–4	
**Haematologic**											
Leukopenia	204	83	183	105	0.066	221	67		248	40	0.005
Neutropenia	183	105	181	107	0.931	229	59		249	39	0.035
Anaemia	281	7	280	8	1.000	279	9		285	3	0.145
Thrombocytopenia	281	7	282	6	1.000	281	7		286	2	0.179
Liver function	287	1	286	2	1.000	288	0		288	0	1.000
Renal function	287	1	288	0	1.000	288	0		288	0	1.000
**Non-haematologic**											
Mucositis	286	2	285	3	1.000	212	76		248	40	<0.0001
Dermatitis	287	1	288	0	1.000	281	7		283	5	0.770
Diarrhoea	282	6	284	4	0.750	288	0		288	0	1.000
Nausea/vomiting	267	21	272	16	0.497	243	45		281	7	<0.0001

Abbreviations: NAC neoadjuvant chemotherapy; IMRT intensity-modulated radiotherapy; CCRT concurrent chemoradiotherapy.

## Discussion

Our study was a large-scale observational study of locoregionally advanced NPC patients treated with additional NAC before CCRT or NAC, followed by IMRT alone. We found that NAC + IMRT alone conferred comparable long-term survival outcomes when compared to adding NAC before CCRT for NPC female patients who were <60 years, or stage T1-2 or stage N0-1, while with less acute toxicities. To our knowledge, this is the first observational study to compare long-term survival and toxicities in locoregionally advanced NPC patients receiving additional NAC to IMRT alone with NAC before CCRT. Accordingly, we can conclude that the omission of CC from the standard treatment did not affect survival outcomes for special subgroups of NPC patients.

CCRT with or without AC is still the first line of treatment recommended by the National Comprehensive Cancer Network because of the improved survival advantages^23^. However, a phase III randomised trial performed by Sun *et al*. revealed that adding TPF-based NAC before CCRT achieved the benefit of failure-free survival for locoregionally advanced NPC with manageable toxicities^24^. A recent meta-analysis showed that the addition of NAC before CCRT significantly increased PFS and OS and was associated with more frequent complications in locoregionally advanced NPC patients^25^. Other previous studies indicated that the addition of NAC before CCRT or IMRT conferred the promising survival outcomes^26–30^. Thence, NAC + CCRT could be an alternative therapy of CCRT for locoregionally advanced NPC. Approximately 63% of patients were unable to receive CC due to toxicities^9^. Thus, the effect of omitting CC on the survival outcomes of locoregionally advanced NPC remains unclear.

Komatsu *et al*. found that NAC followed by RT yielded similar survival outcomes with CCRT in locoregionally advanced NPC patients^18^. Huang *et al*. did a randomised study to compare the efficacy of adding NAC before CCRT versus NAC followed by RT alone, and their results revealed that NAC plus RT alone was not inferior to NAC, followed by CCRT in OS and PFS^17^. Wu *et al*. also found that additional NAC before RT alone provided long-term survival comparable to that with CCRT for locoregionally advanced NPC^19^. A phase III randomised study conducted by Xu *et al*. showed that when compared with CCRT followed by AC, NAC + IMRT and AC obtained similar OS and PFS and less acute toxicities^20^. Liu *et al*. investigated the long-term survival of 256 NPC patients who completed CCRT or NAC plus RT, and their results revealed that NAC plus RT obtained similar long-term survival outcomes and less acute grade 3/4 toxicities^31^. It is important to note that the 2-dimension RT technology was used for NPC patients in all the studies mentioned above. QuYang *et al*. conducted a retrospective study to investigate the efficacy of 94 NPC patients receiving additional NAC to IMRT alone in 302 NPC patients treated with CCRT, and found comparable long-term survival outcomes^32^. A recent retrospective study conducted by Yao *et al*. exhibited that the treatment outcomes in the NAC + RT group were similar to those in the CCRT group, with less acute and later toxicities for 214 patients with ascending-type NPC^33^.

In current matched study, we examined survival over a follow-up time of 86 months; 5-year LRRFS, DMFS, PFS, and OS rates for the cohort of locoregionally advanced NPC patients were 95.4%, 91.8%, 88.2%, and 91.7%, respectively. Compared with NAC followed by CCRT, the addition of NAC before IMRT alone yielded similar LRRFS (96.1% vs. 94.7%, P = 0.201), DMFS (93.7% vs. 89.8%, P = 0.129), OS (93.0% vs. 90.6%, P = 0.362) and worse PFS (91.3% vs. 85.1%, P = 0.024) for these patients. In addition, multivariate analyses indicated that NAC plus IMRT alone were associated with an unfavourable prognostic factor of PFS (P = 0.014). In the subgroup analysis, the 5-year PFS (92.9% vs. 88.4%, P = 0.297; 90.1% vs. 85.6%, P = 0.124; 88.6% vs. 87.2%, P = 0.543; 93.2% vs. 88.3%, P = 0.398) were similar in the two groups for NPC female patients who were <60 years, or T1-2 or N0-1.

The most commonly observed acute adverse events included haematologic and non-haematologic toxicities during the period of NAC and IMRT. Although all patients in this study received prophylactic leukocyte therapy by the use of recombinant granulocyte colony-stimulating factor (GCFS), a few patients still experienced grade 3/4 leukocytopenia and neutropenia during NAC administration and could continue with chemotherapy without delay by receiving GCSF. The incidences of grade 3/4 haematologic and non-haematologic toxicities during the NAC period were comparable in both groups. The acute side effects during the IMRT period revealed a significant decrease of leukocytopenia (23.3% vs. 13.9%, P = 0.005) and neutropenia (20.5% vs. 13.5%, P = 0.035) in the NAC + IMRT group. No statistically significant differences were observed in other haematologic toxicities in both arms. The acute non-haematologic toxicities included mucositis, dermatitis, diarrhoea, and nausea/vomiting during the IMRT period, and were mild to moderate. The incidences of grade 3/4 mucositis and nausea/vomiting were significantly lower in NAC + IMRT alone group than in the NAC + CCRT group (mucositis: 26.4% vs. 13.9%, P < 0.0001; nausea/vomiting: 15.6% vs. 2.3%, P < 0.0001). Liu *et al*. found that the NPC patients who received NAC + RT developed less grade 3/4 mucositis (55% vs. 16%, P < 0.0001), neck dermatitis (31% vs. 16%, P < 0.004) and vomiting (23% vs. 0%, P < 0.0001)^31^. The previously reported incidences of grade 3/4 mucositis during CCRT with a weekly low-dose^34–36^ and tri-weekly high-dose^9,24,37,38^ cisplatin were 31.4–48.9% and 29–62%, respectively. The rate of grade 3/4 mucositis in the present study was slightly less due to the use of IMRT.

This large-scale observational study was conducted by a single centre in an endemic area. Our major limitation was that the results of a single-arm retrospective study provided relatively low power to indicate non-inferior outcomes of NAC + IMRT. This study only evaluated acute treatment-associated toxicities and no late complications. The acute toxicities were assessed according to medical record information. In addition, the NAC regimen and doses were heterogenetic due to the retrospective design. Hence, our results should be regarded as preliminary. Further prospective, randomised, multicentre clinical trials are paramount to be conducted in the future.

## Conclusion

In conclusion, our study showed that NAC + IMRT alone obtained similar survival outcomes with NAC before CCRT for locoregionally advanced NPC patients, and a lower incidence of grade 3/4 acute toxicities. Therefore, the omission of CC did not affect overall survival outcomes. However, further randomised, controlled, multicentre phase III clinical trials are needed to assess the ultimate efficacy and toxicity of NAC + IMRT alone.

## Patients and Methods

### Patient selection

Between May 2008 and April 2014, patients who received treatment in the Department of Radiation Oncology at Zhejiang Cancer Hospital were retrospectively reviewed. The eligible patients met the following criteria: (i) untreated, newly diagnosed locoregionally advanced NPC, (ii) Eastern Cooperative Oncology Group performance status ≤1, (iii) completion of radical IMRT, (iv) received NAC + IMRT with or without CC, and (v) no previous anticancer treatment. This retrospective study was approved by the Medical Ethics Committee and the Institutional Reviewed Board of Zhejiang Cancer Hospital. Each patient had signed an informed consent form.

**Figure 5:**
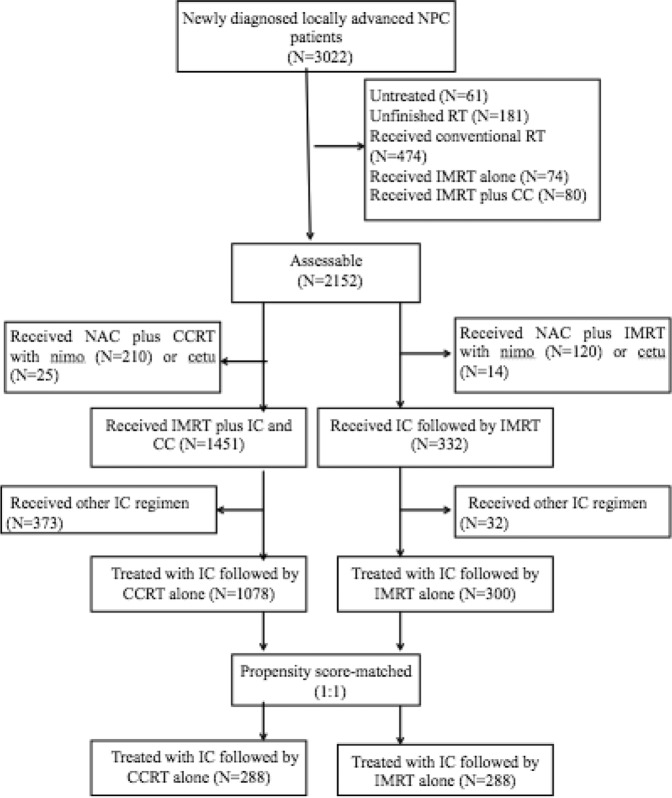
Flowchart of patients. NPC: Nasopharyngeal carcinoma; RT: Radiotherapy. IMRT intensity-modulated radiotherapy; CC: concurrent chemotherapy; CCRT: concurrent chemoradiotherapy; NAC: neoadjuvant chemotherapy; nimo: nimotuzumab; cetu: cetuximab.

The present study was an observational, matched study performed in accordance with the Declaration of Helsinki and good clinical practice guideline. The flowchart of patients is shown in Fig. 5. A total of 3,022 newly diagnosed locoregionally advanced NPC patients were registered at Zhejiang Cancer Hospital. A total of 576 NPC patients, identified by a propensity score-matched method, were enrolled in our study. All patients received NAC combined with definitive IMRT with or without CC.

### Basic examinations

The patients had pre-treatment evaluations that included complete medical histories, physical examinations, haematology and biochemistry profiles, chest radiographs, abdominal sonography, bone scans, magnetic response images of the nasopharynx, and nasopharyngoscopies. All patients were staged according to the 2010 American Joint Committee on Cancer staging system. Tumour histology was classified per the World Health Organization classification.

### IMRT

All patients were immobilised in the supine position with thermoplastic masks. Computed tomography with intravenous contrast (2.5 mm slices from the head to 2 cm below the sternoclavicular joints) were performed for planning. All patients underwent radical IMRT with a simultaneous integrated boost technique that used 6-MV photons with 2–3 weeks after NAC. The delineation of target volumes of NPC during the treatment of IMRT was described previously^39–41^. The prescribed radiation doses were 69 Gy or 72 Gy to planning gross target volume (PGTV)nx, 66 Gy to 69 Gy to PGTVnd, 63 Gy to 66 Gy to planning target volume (PTV)nx, 60 Gy to 63 Gy to PTV1, and 51 Gy to 54 Gy to PTV2, delivered in 30 or 33 fractions. Radiation was delivered once daily, in five fractions per week, for over 6–6.5 weeks for IMRT planning. The dose to organs at risk was limited based on the Radiation Therapy Oncology Group 0225 protocol.

### Chemotherapy

All patients received 1–4 cycles of 3-weekly platinum-based NAC. The available NAC regimens included TPF (docetaxel 60 mg/m^2^/day on day 1, cisplatin 25 mg/m^2^/day on days 1 to 3, and 5-fluorouracil 500 mg/m^2^/day on days 1 to 3), TP (docetaxel 60 mg/m^2^/day on day 1, cisplatin 25 mg/m^2^/day on days 1 to 3), GP regimen (gemcitabine 1,000 mg/m^2^/day on days 1 and 8, cisplatin 25 mg/m^2^/day on days 1–3), and FP (cisplatin 25 mg/m^2^/day on days 1–3, and 5-fluorouracil 500 mg/m^2^/day on days 1–3).

Moreover, 288 NPC patients in this study underwent ≥1 cycle CC with cisplatin (80 mg/m^2^) divided in 3 days. About 349 patients received 2–3 courses of AC with FP (cisplatin 25 mg/m^2^/day on days 1–3, and 5-fluorouracil 500 mg/m^2^/day on days 1–3) regimen three weeks after RT.

### Patient evaluation and follow-up

The assessment of tumour response was performed thrice after the completion of IC, at the end of IMRT, and three months after RT, which was based on MRI and nasopharynx fiberscope according to the Response Evaluation Criteria for Solid Tumors. Systemic chemotherapy adverse effects were graded using the National Cancer Institute Common Toxicity Criteria (NCI CTCAE, version 4.0). In contrast, RT-induced toxicities were scored according to the Acute and Late Radiation Morbidity Scoring Criteria of the Radiation Therapy Oncology Group (RTOG).

All the participants underwent weekly examinations for treatment response and toxicities during the IMRT. Patients were followed-up every three months in the first two years, every six months from the third to the fifth year, and then annually. Each follow-up included careful examination of the nasopharynx and neck nodes by an experienced physician, an MRI scan of the nasopharynx, nasopharynx fiberscope, chest computed tomography radiograph, and abdominal ultrasound were performed three months after the completion of RT and every 6–12 months after that. Additional examinations were performed when it was indicated to evaluate local relapse or distant metastasis.

### Statistical analysis

The primary outcomes of the present study were locoregional relapse-free survival (LRRFS), distant metastasis-free survival (DMFS), PFS, OS, and acute toxicities from NAC and IMRT. OS, LRRFS, DMFS, and PFS were defined as the time from the enrolment date in the trial to the date of death or the date of the last follow-up, the first local or regional relapse, the first distant metastasis occurrence, or the diagnosed evidence of disease progression or the last follow-up, respectively. After relapse or metastasis, patients received salvage therapy as determined by their physicians.

IBM SPSS Statistics version 22.0 was used for all the data analysis. We compared the patients’ characteristics, acute toxicities, and patterns of failure between the two groups by the Chi-square test or Fisher’s exact test. Survival curves were generated using the Kaplan-Meier method. The curves were compared using log-rank tests. Multivariate analysis was performed using Cox regression models to identify significant prognostic factors. Hazard ratios (HRs) and 95% confidence intervals (CIs) were calculated for each prognostic factor. A P < 0.05 was considered statistically significant.

We adjusted potential biases related to treatment with specific therapeutic regimens using a PSM analysis^42,43^. We computed propensity scores for every patient by logistic regression according to sex, age category (<60 years/≥60 years), T-stage, N-stage, clinical stage, NAC regimen, and NAC cycle. PSM was performed using all the above covariates with a one-to-one nearest neighbour matching algorithm at a caliper of 0.02.
